# Fiber and Prebiotics: Mechanisms and Health Benefits

**DOI:** 10.3390/nu5041417

**Published:** 2013-04-22

**Authors:** Joanne Slavin

**Affiliations:** Department of Food Science and Nutrition, University of Minnesota, 1334 Eckles Avenue, St. Paul, MN 55344, USA; E-Mail: jslavin@umn.edu; Tel.: +1-612-624-7234; Fax: +1-612-625-5272

**Keywords:** dietary fiber, prebiotics, fermentation, microbiota, short chain fatty acids, immune function

## Abstract

The health benefits of dietary fiber have long been appreciated. Higher intakes of dietary fiber are linked to less cardiovascular disease and fiber plays a role in gut health, with many effective laxatives actually isolated fiber sources. Higher intakes of fiber are linked to lower body weights. Only polysaccharides were included in dietary fiber originally, but more recent definitions have included oligosaccharides as dietary fiber, not based on their chemical measurement as dietary fiber by the accepted total dietary fiber (TDF) method, but on their physiological effects. Inulin, fructo-oligosaccharides, and other oligosaccharides are included as fiber in food labels in the US. Additionally, oligosaccharides are the best known “prebiotics”, “a selectively fermented ingredient that allows specific changes, both in the composition and/or activity in the gastrointestinal microflora that confers benefits upon host well-bring and health.” To date, all known and suspected prebiotics are carbohydrate compounds, primarily oligosaccharides, known to resist digestion in the human small intestine and reach the colon where they are fermented by the gut microflora. Studies have provided evidence that inulin and oligofructose (OF), lactulose, and resistant starch (RS) meet all aspects of the definition, including the stimulation of *Bifidobacterium*, a beneficial bacterial genus. Other isolated carbohydrates and carbohydrate-containing foods, including galactooligosaccharides (GOS), transgalactooligosaccharides (TOS), polydextrose, wheat dextrin, acacia gum, psyllium, banana, whole grain wheat, and whole grain corn also have prebiotic effects.

## 1. Introduction

### 1.1. Dietary Fiber

The term “dietary fiber” was coined in 1953, but the health benefits of high fiber foods have been long appreciated [1]. In 430 BC, Hippocrates described the laxative effects of coarse wheat in comparison with refined wheat [1]. In the 1920s, J.H. Kellogg published extensively on the attributes of bran [1], claiming it increased stool weight, promoted laxation, and prevented disease. Dietary fiber was researched throughout the 1930s, and then forgotten until the 1970s. 

Denis Burkitt is usually credited with re-popularizing the idea that dietary fiber protects against development of Western diseases, including diabetes, cardiovascular disease, colon cancer, and obesity [1]. Since that time, research continues on defining fiber, measuring fiber, and determining the health benefits of fiber consumption. Prospective, cohort studies show clearly that high fiber intakes are linked to less cardiovascular disease. Data on obesity, diabetes, and cancer are more mixed. Dietary fiber is listed on the Nutrition Facts panel on food products and nutrient content claims are allowed for good (2.5 g fiber) and excellent (5.0 g fiber) sources of fiber.

### 1.2. Prebiotics

Prebiotics were first defined as “nondigestible food ingredients that beneficially affect the host by selectively stimulating the growth and/or activity of one or a limited number of bacteria in the colon, thus improving host health” [2]. This definition was later refined to include other areas that may benefit from selective targeting of particular microorganisms [3]: “a selectively fermented ingredient that allows specific changes, both in the composition and/or activity in the gastrointestinal microflora, that confer benefits.” Lactobacilli and bifidobacteria are the usual target genera for prebiotics; changes in bifidobacteria are more likely to be seen compared to lactobacilli. This may be due to the fact that more bifidobacteria usually reside in the human colon than lactobacilli, and they exhibit a preference for oligosaccharides. 

Although all prebiotics are fiber, not all fiber is prebiotic. Classification of a food ingredient as a prebiotic requires scientific demonstration that the ingredient [2]:

- Resists gastric acidity, hydrolysis by mammalian enzymes, and absorption in the upper gastrointestinal tract;
- Is fermented by the intestinal microflora;
- Selectively stimulates the growth and/or activity of intestinal bacteria potentially associated with health and well-being.

The concept of prebiotics is relatively new [2]. Roberfroid *et al.* [4] updated the definition of prebiotics, based on and ILSI Europe task force. Foods high in prebiotics have been consumed since prehistoric times. Archaeological evidence from dry cave deposits in the northern Chihuahuan Desert show intensive utilization of desert plants that were high in inulin [5]. Analysis of well-preserved coprolites suggest that dietary intake of inulin was about 135 g/day for the typical adult male hunter-forager. Prebiotics occur naturally in foods such as leeks, asparagus, chicory, Jerusalem artichokes, garlic, onions, wheat, oats, and soybeans [6]. Consumption in typical US and European diets has been estimated to be several grams per day. The caloric value of nondigestible oligosaccharides has been estimated between 1 and 2 kcal/g [7]. Some known prebiotics (inulin) are low digestible carbohydrates and are associated with impaired gastrointestinal tolerance, especially when consumed in large quantities [8,9] while other prebiotic fibers (e.g., wheat dextrin, polydextrose) exhibit high gastrointestinal tolerability (30–45 g per day) [10].

## 2. Colonic Microflora and Fermentation

An important mechanism of action for dietary fiber and prebiotics is fermentation in the colon and changes in gut microflora. The human large intestine is one of the most diversely colonized and metabolically active organs in the human body [11]. Up to 1000 different species of bacteria reside in the colon with microbial populations comprising approximately 10^11^–10^12^ cfu/g of contents. The colonic environment is favorable for bacterial growth due to its slow transit time, readily available nutrients, and favorable pH [12]. Generally, bacteria having an almost exclusive saccharolytic metabolism (*i.e.*, no proteolytic activity) can be considered potentially beneficial. Such a metabolic profile is typical for lactobacilli and bifidobacteria. Mapping the diversity of and interactions among the human intestinal microflora has led to the initiation of the Human Gut Microbiome Initiative (HGMI) [13], an effort to identify this bacterial ecosystem.

Together with the gut immune system, colonic and mucosal microflora contributes significantly to the barrier that prevents pathogenic bacteria from invading the gastrointestinal (GI) tract. The intestinal flora salvages energy through fermentation of carbohydrates not digested in the upper gut. The main substrates are endogenous (e.g., mucus) and dietary carbohydrates that escape digestion in the upper GI tract. These include resistant starch, non-starch polysaccharides (e.g., celluloses, hemicelluloses, pectins, and gums), non-digestible oligosaccharides, and sugar alcohols. The main fermentation pathway generates pyruvate from hexoses in the undigested carbohydrate. Colonic bacteria use a range of carbohydrate hydrolyzing enzymes to produce hydrogen, methane, carbon dioxide, SCFAs (mainly acetate, propionate and butyrate), and lactate. Certain colonic bacteria generate energy from these fermentation products. Dietary components that stimulate fermentation lead to an increase in bacterial mass and consequently fecal mass and, thus have a stool bulking effect. It is estimated that about 30 g of bacteria are produced for every 100 g of carbohydrate that is fermented. 

At both the colonic and systemic levels, fermentation and especially SCFA production play an integral role. Colonic epithelial cells preferentially use butyrate as an energy source, even when competing substrates such as glucose and glutamine are available. Butyrate is considered a key nutrient determining the metabolic activity and growth of colonocytes and may function as a primary protective factor against colonic disorders, although data on this topic are conflicting [14]. SCFAs are water-soluble and are absorbed into the blood stream. The brain, muscles, and tissues metabolize acetate systemically whereas propionate is cleared by the liver and may lower the hepatic production of cholesterol by interfering with its synthesis. Transport to and further metabolism of SCFAs in the liver, muscle, or other peripheral tissues is thought to contribute about 7%–8% of host daily energy requirements [12]. Fermentation and SCFA production also inhibit the growth of pathogenic organisms by reducing luminal and fecal pH. Low pH reduces peptide degradation and the resultant formation of toxic compounds such as ammonia, amines, and phenolic compounds, and decreases the activity of undesirable bacterial enzymes. 

Overall, a number of factors influence the composition of the microflora. These include changes in physiological conditions of the host (e.g., age stress, health status), composition of the diet, and environmental circumstances (e.g., antibiotic therapy, hygiene with antiseptics, *etc.*) [15]. Recognition of the health-promoting properties of certain gut microorganisms has encouraged dietary-based modulation of the human intestinal microflora towards a more beneficial composition and metabolism. Prebiotic fiber, a class of fiber that may act to beneficially alter the colonic microflora, has generated intense scientific, consumer, and regulatory debate since it was first defined in the mid-1990s. A summary of a workshop on prebiotics and the health benefits of fibers was recently published [16] and concluded that additional research is needed to define the relationship between the consumption of prebiotics and improvement of human health.

## 3. Current Understanding of Fiber

Fiber is defined differently throughout the world. Some definitions are based on analytical methods for isolating fiber, while there is a move to define fiber on a physiological basis. Traditonally, fiber was measured as chemical components, such as cellulose, hemicellulose, pectin, and lignin, the only noncarbohydrate component of fiber. Currently the United States relies on an “analytical approach” to determine what is or is not considered fiber for purposes of listing fiber content on food labels. In 2001 the Institute of Medicine (IOM) developed the following set of working definitions for fiber in the food supply [17]:

*Dietary fiber* consists of nondigestible carbohydrates and lignin that are intrinsic and intact in plants.

*Functional fiber* consists of isolated, nondigestible carbohydrates that have beneficial physiological effects in humans.

These definitions recognize the diversity of nondigestible carbohydrates in the food supply. This definition has yet to be formally adopted by the U.S. Food and Drug Administration (FDA), but it includes plant, animal, and manufactured fiber sources that exhibit beneficial physiological effects in humans.

Progress has been slow on agreeing to a universal definition of dietary fiber [18]. Codex Alimentarius Commision in 2009 published a dietary fiber definition [18]. Some of the outstanding issues about that definition were debated at the Fahouny Fiber Symposium: (1) Inclusion or exclusion of undigestible carbohydrates with degrees of polymerization (DP) in the range of 3 and 9 was left to the discretion of national authorities; (2) The absence of a list of beneficial physiological effects and appropriate criteria for their substantiation; (3) The analytical methodology by which fiber in food was to be quantified.

Traditionally, dietary fiber was classified according to its solubility in an attempt to relate physiological effects to chemical types of fiber [17]. Soluble fibers were considered to have benefits on serum lipids, while insoluble fibers were linked with laxation benefits. This division of soluble and insoluble fiber is still used in nutrition labeling. However, despite these commonly used generalizations, scientific evidence supporting that soluble fibers lower cholesterol and insoluble fibers increase stool weight is inconsistent. Resistant starch and inulin, both soluble fibers, do not appear to lower blood cholesterol, and the effect of insoluble fiber on stool weight is highly variable. In addition, many fiber sources are mostly soluble but still enlarge stool weight, such as oat bran and psyllium.

Most commonly consumed foods are low in dietary fiber. Generally, accepted servings of food contain from 1 to 3 g of fiber per serving. Higher fiber contents are found in foods such as whole grain cereals, legumes, and dried fruits. Other fiber sources include over-the-counter laxatives containing fiber, fiber supplements, and fiber-fortified foods. 

The Nutrition Facts label is based on 25 g of fiber recommended daily for a 2000 calorie diet. Americans typically consume about half of the recommended amounts of fiber each day (about 15 g/day) [17]. Flours, grains, and potatoes are the most popular sources of fiber in the American diet; while fruits, legumes, and nuts are the least popular sources [17]. 

Foods that are high in fiber, whole grains, vegetables, fruits, and legumes contain more than just fiber. These co-passengers with fiber may provide the protective health properties of fiber, rather than the fiber itself [19]. Also, additional properties of fiber, such as viscosity and fermentability, may be more important characteristics in terms of physiological benefits. Viscous fibers are those that have gel-forming properties in the intestinal tract, and fermentable fibers are those that can be metabolized by colonic bacteria. In general, soluble fibers are more completed fermented and have a higher viscosity than insoluble fibers. However, not all soluble fibers are viscous (e.g., partially hydrolyzed guar gum and acacia gum) and some insoluble fibers may be well fermented (Table 1).

**Table 1:** Classification of fibers based on four characteristics [20].

Fibers	Classification
Dietary Fiber	Lignin
	Cellulose
	B-glucans
	HemicellulosesPectins
	Gums
	Resistant Starch
Soluble Fibers	B-glucans
	Gums
	Wheat dextrin
	Psyllium
	Pectin
	Inulin
Fermentable Fiber	Wheat dextrin
	Pectins
	B-glucans
	Guar gum
	Inulin
Viscous Fibers	Pectins
	B-glucans
	Some gums (e.g., guar gum)
	Psyllium
Functional Fiber	Resistant dextrins
	Psyllium
	Fructooligosaccharides
	Polydextrose
	Isolated gums
	Isolated resistant starch
Insoluble Fibers	Cellulose
	Lignin
	Some pectins
	Some hemicelluloses
Non-fermentable Fibers	Cellulose
	Lignin
Non-viscous Fibers	Polydextrose
	Inulin

## 4. Health Benefits of Fiber

Evidence-based reviews use an accepted hierarchy to examine the body of evidence for a clinical nutrition question [21]. Typically double-blind, randomized, controlled trials are the gold standard for clinical evidence. Well-conducted intervention studies in target populations are also well regarded in evidence-based reviews. Prospective, cohort epidemiological studies provide important support for diet and disease relationships. Lower level knowledge examples are clinical cases and expert opinion.

Animal studies and *in vitro* studies provide important clues for mechanisms for a relationship between disease and dietary exposure, but are typically not included in evidence-based reviews. In dietary fiber research, much of our information on fermentation of fibers is based on in vitro models of fermentation. These data help us compare fibers, but need to be tested in human clinical trials to support their use in clinical nutrition.

Only human data are included in this review of the health outcomes of dietary fiber and prebiotics. Fecal samples are not typically collected in prospective, cohort studies, so few relationships between intake of prebiotics and health outcomes are not available. Accepted protocols to measure the prebiotic potential of fibers or oligosaccharides do not exist, so reviewed studies include those found where a fiber or oligosaccharide were fed to healthy human subjects and changes in microbiota measured. Studies in diseased populations are not included in this review, nor are studies in infants.

### 4.1. Cardiovascular Disease

The AI level of 14 g of fiber per 1000 kcals of energy consumed is based on protection against cardiovascular disease (CVD); so the data for this relationship are strong [17]. Epidemiologic studies suggest that adequate fiber intake consistently lowers the risk of CVD and coronary heart disease (CHD), primarily through a reduction in low density lipoprotein (LDL) levels. The results of randomized clinical trials are inconsistent, but suggest that fiber may play a beneficial role in reducing C-reactive protein levels, apolipoprotein levels, and blood pressure, all of which are biomarkers for heart disease. Water-soluble fibers (specifically, beta-glucan, psyllium, pectin, and guar gum) were most effective for lowering serum LDL cholesterol concentrations, without affecting high density lipoprotein (HDL) concentrations. In the U.S., there are accepted health claims for the ability of oats, barley, and psyllium to lower blood lipids. Other soluble fibers, glucans and pectins, have recognized ability to lower blood lipids and the regulations in individual countries determine labeling and claims.

### 4.2. Type II Diabetes and Glycemic Control

There are many theories surrounding the relationship between fiber intake and type II diabetes. For example, regularly consuming the recommended amount of fiber has the potential to attenuate glucose absorption rate, prevent weight gain, and increase the load of beneficial nutrients and antioxidants in the diet, all of which may help prevent diabetes. 

Numerous large-scale cohort studies support a strong inverse relationship between dietary fiber consumption and development of type II diabetes. A multi-ethnic cohort followed 75,000 people for 14 years. People who ate more than 15 g of fiber per day had significantly lower diabetes risk [22]. People who ate high amounts of insoluble fiber (more than 17 g/day) or cereal fiber (more than 8 g/day) had less type II diabetes risk than people who had lower intakes while soluble fiber intake was not associated with diabetes risk [23]. 

Intervention studies provide inconsistent results. For instance, compared to a 5-week control diet, 5 weeks of oat beta-glucan (5 g) significantly reduced postprandial glucose and insulin responses, while 5 weeks of barley beta-glucan (5 g or 10 g) did not [24]. Nazare *et al.* [25] found significant reductions in glucose and insulin when fiber was added to a standard breakfast. Many acute intervention trials fail to find a relationship between fiber intake and post-prandial glucose response [26]. 

### 4.3. Laxation and Regularity

It is well recognized that fiber is important for normal laxation. This is due primarily to the ability of fiber to increase stool weight. The increased weight is due to the physical presence of the fiber, water held by the fiber, and increased bacterial mass from fermentation. Larger and softer stools increase the ease of defecation and reduce transit time through the intestinal tract, which may help to prevent or relieve constipation. In general, cereal fibers are the most effective at increasing stool weight. Wheat bran is considered the “gold standard” when it comes to fecal bulking, since no other fiber or laxative has been shown to be as effective [27]. Inulin, although extensively fermented, has little effect on stool weight [28], with less than a 1 g/ increase in stool weight with each g fiber fed as inulin.

The effect of fiber and low digestible carbohydrates on gastrointestinal tolerance is a concern. Not all fibers have the same effect on tolerance; fructo-oligosaccharides can cause symptoms with low doses (10 g) [29] while other fibers, such as polydextrose and resistant starch have been consumed at doses up to 50 g without symptoms [30]. It is likely that fast and complete fermentation in the upper gut is linked to GI intolerance.

### 4.4. Appetite Control

Multiple mechanisms describe how fiber influences satiation and satiety [31]. Greater satiation may be a product of the increased time required to chew certain fiber-rich foods. Increased time chewing promotes saliva and gastric acid production, which may increase gastric distention. Some soluble/viscous fibers bind water, which also may increase distention. Stomach distension is believed to trigger afferent vagal signals of fullness, which likely contributes to satiation during meals and satiety in the post-meal period.

Furthermore, certain fibers may slow gastric emptying and decrease the rate of glucose absorption in the small intestine. When glucose is released slowly, the insulin response may also be blunted. Slow, steady post-prandial glucose and insulin responses are sometimes correlated with satiation and satiety.

As food moves through the upper and lower gastrointestinal (GI) tract, various satiety-related hormones are released and signals are sent to the brain. Many of these gut hormones (*i.e.*, ghrelin, polypeptide YY, glucagon-like peptide) are thought to regulate satiety, food intake, and overall energy balance [32]. 

Recently in a randomized, double-blind, placebo-controlled clinical study in 100 overweight healthy Chinese adults investigated the effect of different dosages of dietary supplementation with wheat dextrin, on satiety over time [33]. Subjects were randomized by body mass index and energy intake and assigned to receive either placebo or 8, 14, 18, or 24 g/day of wheat dextrin (*n* = 20 volunteers per group). On days −2, 0, 2, 5, 7, 14, and 21, short-term satiety (up to 120 min) was evaluated with a visual analog scale, and hunger feeling status was assessed with Likert scale. Wheat dextrin increased short-term satiety, which was time and dosage correlated. The hunger feeling status was evaluated for 21 days. The hunger feeling decreased significantly from day 5 to the end of the evaluation for the group 24 g and from day 7 for the groups 14 and 18 g. By day 5, the group 24 g showed significantly longer time to hunger between meals compared with placebo. The caloric intake per day was evaluated during a 9 week study. A significant decrease in caloric intake was seen from week 2 to the end of the 9 week study for the groups 14 g, 18 g and 24 g of wheat dextrin. 

### 4.5. Body Weight

Prospective cohort studies report that people who consume higher amounts of fiber weigh less than people who consume lesser amounts [17]. One study reported that in a 20-month period, every 1 g increase in total fiber consumed per day, decreased body weight by 0.25 kg [34]. 

Fiber intake associates with other beneficial lifestyle factors, such as fruit and vegetable intake and exercise habits. Diets that are high in fiber are typically lower in fat and energy density, both of which are helpful for maintaining a healthy body weight. Howarth *et al.* [35] summarized the results of more than 50 intervention studies that had assessed relationships among energy intake, body weight, and fiber intake. They estimated that increasing fiber intake by 14 g per day was associated with a 10% decrease in energy intake and a 2 kg weight loss over about a 4-month period. The observed changes in energy intake and body weight occurred without regard to the fiber’s source as a naturally high-fiber food or a functional fiber supplement.

The involvement of gut microbiota in the regulation of host energy homeostasis was suggested by studies reporting that obese people were shown to have lower Bacteroidetes and more Firmicutes in their distal gut than lean control individuals, alterations that were abolished after 52 weeks of diet-induced weight loss [36]. Changing gut microflora may be more difficult in free-living individuals and longterm consequences of changes in gut microflora are unknown [37].

### 4.6. Cancer

In the 1970s, many reports suggested that increased colorectal cancer prevalence was a result of low-fiber diets. These assumptions were predominantly based on differences in colorectal cancer rates among nations and regions with high- and low-fiber intakes; this type of data clearly lacks causal evidence. Several large-scale studies, including some intervention trials, have suggested fiber intake is not associated with overall risk for colorectal cancer. For example, the 8-year Polyp Prevention Trial (PPT) evaluated the effects of a high-fiber (18 g/1000 kcal), high fruit and vegetable, and low-fat diet on the recurrence of adenomatous polyps in the colon [38]. This study failed to show an effect of diet on adenoma recurrence after 8 years of follow-up. The lack of relationship between high-fiber diet interventions and colorectal cancer risk may be authentic, or it may be a product of the long latency period for colorectal cancer development. 

### 4.7. Prebiotic Effect and SCFA Production

Fermentable fibers may provide a number of health benefits by altering the composition of the intestinal flora. Prebiotics are non-digestible substances that provide a beneficial physiological effect to the host by selectively stimulating the favorable growth or activity of a limited number of indigenous bacteria. This generally refers to the ability of a fiber to increase the growth of bifidobacteria and lactobacilli, which are considered beneficial to human health. Benefits of prebiotics include improvement in gut barrier function and host immunity, reduction of potentially pathogenic bacteria subpopulations (e.g., clostridia), and enhanced SCFA production. 

Inulin, oligofructose, and FOS have been extensively studied as prebiotics, and have been shown to significantly increase fecal bifidobacteria at fairly low levels of consumption (5–8 g per day). A very-long chain inulin extracted from globe artichoke (*Cynara scolymus*) had pronounced prebiotic effect in human subjects, but was well tolerated [39]. Fruit and vegetable shots containing Jerusalem artichoke inulin were given to 66 healthy volunteers. Fluorescent in situ hybridization was used to monitor populations of microbiota [40]. Inulin from Jerusalem artichoke was found to have prebiotic potential.

The ability to favorably alter the intestinal microflora has been demonstrated by a number of other fiber and plant food sources (Table 2). Acacia gum was shown to produce a greater increase in bifidobacteria and lactobacilli than an equal dose of inulin, and resulted in fewer gastrointestinal side effects, such as gas and bloating [41]. Polydextrose consumption resulted in a dose-dependent decrease in bacteroides, as well as an increase in lactobacilli and bifidobacteria [42,43]. Wheat dextrin has also been shown to increase lactobacilli and reduce *Clostridium perfringens* and increase bifidobacteria [44]. In a study with 40 female subjects, wheat dextrin supplementation (8 g per day) for fourteen days not only increased bacteroides, the predominant beneficial saccharolytic genus of a normal gut flora but also decreased the numbers of pathogenic bacteria. Psyllium was found to have prebiotic potential in a small (*n* = 11) study in women [45].

**Table 2:** Human studies with fibers that show prebiotic effects.

Treatment	Prebiotic effects	References
Wheat Dextrin	Increased bacteroides Decreased *Clostridium perfringens*	[44]
Inulin	Bifidogenic	[39,40]
GOS	Bifidogenic	[45]
Acadia gum	Bifidogenic	[34]
Psyllium	Prebiotic potential	[38]
Polydextrose	Bifidogenic	[35,36]
WG breakfast cereal	Prebiotic potential	[38,40]
Banana	Fecal microbiota	[41]

Whole-grain wheat breakfast cereal had a prebiotic effect, while wheat bran did not in a cross-over study with 31 subjects [46]. A maize-based whole grain breakfast cereal mediated a bifidogenic modulation of the gut microbiota, suggesting prebiotic activity [47]. Mitsou *et al.* [48] measured the effect of banana consumption (2 per day) on microbiota. Increases in bifidobacteria were noted, although the study used plate counting as the method to measure bifidobacteria. It should be noted that there are no accepted standard protocols for measurement of microbiotic activity in fecal samples so wide variations are found in sample size, fiber dose, study duration, and method to collect fecal samples and quantitate microbiota [16].

A range of carbohydrates was evaluated for their ability to be utilized by lactobacilli and bifidobacteria [49]. Galacto-oligosaccharides (GOS) and lactulose were shown to support the most favorable growth characteristics, while poor growth was shown with inulin, maltodextrin, and polydextrose. Mixtures of short chain oligosaccharides and inulin showed more growth. 

Fermentable fibers that don’t meet the definition for prebiotics still provide health benefits via production of SCFAs. The three most abundant SCFAs are acetate, propionate, and butyrate, each of which exerts unique physiological effects. Of these, butyrate is considered the most beneficial in terms of colonic health. Butyrate is the preferred energy source for colonic epithelial cells, and promotes normal cell differentiation and proliferation. SCFAs also help regulate sodium and water absorption, and can enhance absorption of calcium and other minerals. In addition, SCFAs act to lower colonic pH, which can inhibit growth of potential pathogens and promote the growth of beneficial bacteria such as bifidobacteria and lactobacilli. Different fibers vary in the amounts and ratio of SCFA produced, as well as in the rate of production. Fibers that are fermented quickly may lead to excessive gas production and bloating, so dose is an important consideration. Fermentation pattern may be related to the molecular weight, chain length, and structure of the fiber. Short chain molecules, such as FOS, are generally fermented more rapidly than larger, longer chain molecules such as acacia gum and PHGG.

### 4.8. Immune Function and Inflammation

Some fibers may also play a role in improving immune function via production of SCFAs. In animal studies, addition of SCFAs to parenteral feeding increases T helper cells, macrophages, and neutrophils, and increased cytotoxic activity of natural killer cells. There is also some evidence of increased resistance to illness or infection with fiber intake. Oligofructose consumption was found to reduce febrile illness associated with diarrhea or respiratory events, and reduce antibiotic use in infants [50]. Certain fibers, such as β-glucans, have been shown to interact with immune cells, and can therefore stimulate the immune system directly. Soluble, non-viscous fiber may also be potentially useful in alleviating symptoms of inflammatory conditions, such as irritable bowel syndrome (IBS). In particular, partially hydrolyzed guar gum has been shown to improve abdominal pain and bowel habits better than wheat bran and qualitative scores of epithelial injury and inflammation compared to control [51]. 

Higher fiber intakes have been linked with lower mortality, particularly from circulatory, digestive and non-CVD/non-cancer inflammatory diseases [52]. Of course when fiber is measured with food frequency instruments in epidemiological studies, all the co-passengers with fiber are also captured in the exposure. Biomarkers that change with fiber intakes, in particular short chain fatty acids and microbiota, have been speculated as important factors.

## 5. Health Benefits of Prebiotics

The health outcome data for prebiotic intake is substantially more limited than for dietary fiber. However, it has been suggested that prebiotic intake may:

- Reduce the prevalence and duration of infectious and antibiotic-associated diarrhea;
- Reduce the inflammation and symptoms associated with inflammatory bowel disease;
- Exert protective effects to prevent colon cancer;
- Enhance the bioavailability and uptake of minerals, including calcium, magnesium, and possibly iron;
- Lower some risk factors for cardiovascular disease; and
- Promote satiety and weight loss and prevent obesity.

## 6. Immunity and Inflammation

### Infectious Diarrhea

In a study of 244 healthy subjects traveling to high- or medium-risk destinations for traveler’s diarrhea, 10 g/day inulin ingested for 2 weeks prior to travel and 2 weeks during travel reduced the prevalence of diarrhea as well as less severe attacks of diarrhea [53]. Either 5.5 g/day GOS or placebo (maltodextrin) was consumed 1 week prior to travel and for the duration of travel to a country with a low or high risk for travelers’ diarrhea [54]. Significant differences (all *p* < 0.05) were observed between the GOS and placebo group in the incidence and duration of travelers’ diarrhea; there were similar findings for abdominal pain and in an overall quality of life assessment.

Intake of a mixture of FOS and inulin has also produced significant reductions in disease severity indices, reduction in pro-inflammatory immune markers, and a reduction in calprotectin, an abundant neutrophil protein found in both plasma and stool that is markedly elevated in patients with inflammatory bowel disease [55]. The efficacy of GOS to change the colonic microflora and improving symptoms in 44 patients with Rome II positive IBS was investigated [56]. GOS significantly enhanced fecal bifidobacteria at 3.5 g/day (*p* < 0.005) and 7 g/day (*p* < 0.001). GOS at 3.5 g/day also significantly changed (all *p* < 0.05) stool consistency, improved flatulence, bloating, composite score of symptoms, and subjective global assessment scores (SGA). GOS at 7 g/day, significantly improved SGA and anxiety scores (both *p* < 0.05). 

Prebiotics may be potential chemopreventative agents based on the observation that health-promoting bacteria such as bifidobacteria do not produce carcinogenic or genotoxic compounds, but instead produce SCFAs, which might be protective. A study of 10 g/day short-chain FOS in adenoma and adenoma-free patients resulted in more positive biomarkers in the adenoma-free patients [57]. Six-month randomized, phase II chemoprevention trial of 76 subjects with previously resected colon cancer or multiple/advanced colorectal adenomas found no effect of 6 g bid FOS-enriched inulin (ORAFTI^®^ Synergy1) on the number of aberrant crypt foci [58].

## 7. Bioavailability and Uptake of Calcium

Studies have shown enhancement of calcium absorption with prebiotic intake, mainly fructans. A 12-month study of 100 adolescents ingesting 8 g/day short- and long-chain inulin fructans showed a significant increase in calcium absorption that led to greater bone mineral density [59]. Daily consumption of cereal containing a combination of short- and long-chain fructo-oligosaccharides (9 g/day) as part of a controlled diet did not benefit calcium absorption or retention in adolescent girls [60]. The benefits of FOS on calcium metabolism may be difficult to see in calcium-replete individuals.

## 8. Cardiovascular Disease

Despite consistent evidence from prospective epidemiologic studies that dietary fiber exerts a protective effect against cardiovascular disease (CVD), the components of dietary fiber that exert this effect are undefined. The DRI Committee concluded that cereal fibers are most effective [61]. Soluble and viscous fibers appear to favorably alter biomarkers of CVD, including low-density lipoprotein cholesterol (LDL-C) and C-reactive protein (CRP). Whether isolated, functional fibers protect against CVD is unclear, although US FDA allows health claims for oats, barley, and psyllium [17].

A double-blind, randomized, placebo-controlled study examined the lipid-modifying ability of 10 g/day inulin/FOS administered for 6 months to 17 normolipidemic participants who consumed their normal diet and did not modify their habits [62]. Compared with placebo, inulin/FOS had no effect on plasma triacylglycerol concentrations and hepatic lipogenesis and induced only a nonsignificant trend for reduced plasma total and LDL-C levels and increased HDL-C concentration. As oligosaccharides are not viscous fibers, it is unlikely that they are particularly good at decreasing absorption of dietary cholesterol. Alternative mechanisms, such as increased production of short chain fatty acids, particularly propionate, are more likely mechanisms if oligosaccharides alter lipid metabolism.

## 9. Obesity, Satiety, and Weight Loss

Studies of lean and obese mice suggest that the gut microbiota affects energy balance by influencing the efficiency of calorie harvest from the diet, and how this harvested energy is utilized and stored [63]. To address how host genotype, environmental exposures, and host adiposity influence the gut microbiome, the fecal microbial communities of adult female monozygotic and dizygotic twin pairs concordant for leanness or obesity, and their mothers were characterized [64]. Obesity was associated with phylum-level changes in the microbiota, reduced bacterial diversity, and altered representation of genes and metabolic pathways. 

A randomized, double-blind, placebo-controlled trial was conducted to examine the effects of FOS supplementation on body weight and satiety hormone concentrations in overweight and obese adults [65]. Forty-eight healthy adults with a body mass index (in kg/m^2^) > 25 received 21 g FOS/day or placebo (maltodextrin) for 12 weeks. There was a reduction in body weight of 1.03 ± 0.43 kg with FOS supplementation, whereas the control group experienced an increase in body weight of 0.45 ± 0.31 kg over 12 weeks (*p* = 0.01). A lower area under the curve (AUC) for ghrelin (*p* = 0.004) and a higher AUC for peptide YY (PYY) with FOS. Suppressed ghrelin and enhanced PYY may contribute in part to the reduction in energy intake. Similar results were obtained in a randomized, double-blind, parallel, placebo-controlled trial of 10 healthy adults that received either 16 g prebiotics/day or 16 g dextrin maltose/day for 2 weeks [66]. Prebiotic treatment increased breath-hydrogen excretion (a marker of gut microbiota fermentation) by approximately 3-fold and lowered hunger rates. Prebiotics increased plasma glucagon-like peptide 1 and peptide YY concentrations, whereas postprandial plasma glucose responses decreased after the standardized meal. Hess *et al.* [67] found that short-chain FOS was extensively fermented in human subjects, but had no effect on satiety or food intake. Recently in a study in overweight adults, wheat dextrin demonstrated a progressive and significant increase satiety, and decrease in hunger feeling at doses 8 to 24 g per day which was time and dosage correlated [68]. 

## 10. Prebiotic Claims

Many countries have no requirement for pre-market approval of prebiotics, because there is no established or implemented system for health claims, although scientific substantiation should be available on request by authorities. The following countries have specific positions and/or policies regarding the use of and claims that can be made for prebiotics.

### 10.1. United States

The Dietary Guidelines for Americans 2010 Committee (DGAC) completed a non-Nutrition Evidence Library (NEL) review of systematic reviews published since 2004 on probiotics, prebiotics, and health [69]. The DGAC believes that the gut microflora do play a role in health and recognizes that consumer interest in altering the microflora is high. Additionally, the DGAC believes that investigation of the gut microflora is an important emerging area of research. However, insufficient evidence was available for the DGAC committee to make dietary recommendations for Americans regarding either prebiotics or probiotics. The DGAC notes that although not all dietary fibers are prebiotics, all prebiotics are dietary fibers. Therefore, the recommended intakes of dietary fiber can provide prebiotics to the diet. In conclusion, the DGAC suggest that foods high in prebiotics (wheat, onions, garlic), should be consumed, as well as food concentrated in probiotics (yogurt).

### 10.2. European Food Safety Authority (EFSA)

Currently, it is the opinion of the EFSA panel that it is not possible to define the exact numbers of different bacterial groups that constitute a ‘normal’ microflora and that an increase in the number of specific microorganisms or any group of microorganisms, including lactobacilli and/or bifidobacteria, is in itself a proven beneficial physiological effect. EFSA has recently published a guidance document focused on two key issues regarding the substantiation of health claims related to the gastro-intestinal tract and immune system (*i.e.*, claimed effects which are considered to be beneficial physiological effects and studies/outcome measures which are considered to be appropriate for the substantiation of health claims) [70].

### 10.3. World Health Organization/Food and Agriculture Organization

Recognizing the possible beneficial effect of prebiotics in food, the Food and Agriculture Organization of the United Nations (FAO) convened a Technical meeting to start work on the evaluation of the functional and health properties of prebiotics [71]. A prebiotic is defined by the FAO as “a non-viable food component that confers a health benefit on the host associated with modulation of the microbiota.” At the Technical meeting, a group of international experts agreed on guidelines and recommended criteria and methodology for systematically approaching the evaluation of prebiotics for safe use in food. It was recommended that a full expert consultation be convened under the auspices of FAO.

## 11. Conclusion

Dietary fibers exhibit a diverse range of physiochemical properties and corresponding physiological effects. The role of fiber in health has extended far beyond improved laxation, and includes benefits on risk factors for cardiovascular disease, weight management, immune function, and colonic health. However, it is clear that not all fibers are equal in terms of the types and extent of health benefits they provide. Characteristics such as solubility, fermentability, and viscosity are important determinants of the effect the fiber will have in the body. Fibers with prebiotic properties can also be recommended as part of fiber intake, although studies are lacking on the benefits of prebiotic intake to healthy individuals. Due to the variability of fiber’s effects in the body, it is important to consume fiber from a variety of sources. Since fiber intakes around the world are less than half of recommended levels, increasing fiber consumption for health promotion and disease prevention is a critical public health goal.
